# β-mannanase supplementation in diets reduced in 85 kcal metabolizable energy/kg containing xylanase-phytase improves gain to feed ratio, nutrient usage, and backfat thickness in finisher pigs

**DOI:** 10.3389/fvets.2023.1144692

**Published:** 2023-03-16

**Authors:** Jansller Luiz Genova, Paulo Evaristo Rupolo, Liliana Bury de Azevedo, Daniela Henz, Silvana Teixeira Carvalho, Marcos Kipper, Giovana de Arruda Castelo Gonçalves, Hellen Lazarino Oliveira Vilela, Tiago Junior Pasquetti, Newton Tavares Escocard de Oliveira, Andrei Roberto Manelli Dietrich, Paulo Levi de Oliveira Carvalho

**Affiliations:** ^1^Animal Science Department, Universidade Federal de Viçosa, Viçosa, MG, Brazil; ^2^Animal Science Department, Universidade Estadual do Oeste do Paraná, Marechal Cândido Rondon, PR, Brazil; ^3^Elanco Animal Health Incorporated Company, São Paulo, SP, Brazil; ^4^Animal Science Department, Universidade Estadual de Mato Grosso do Sul, Aquidauana, MS, Brazil

**Keywords:** blood profile, carcass-meat traits, digestibility, exogenous enzymes, fecal microbiome, pig performance

## Abstract

This study aimed to assess the effects of β-mannanase supplementation in metabolizable energy (ME)-reduced diets containing xylanase-phytase on performance, fecal score, blood biochemical and immunological profile, apparent total tract digestibility (ATTD), digesta passage rate, fecal microbiome, carcass traits and meat quality in finisher pigs (*n* = 40 entire male hybrid, 26.0 ± 0.9 kg) randomly assigned to 1 of 4 dietary treatments: a control diet containing isolated phytase and xylanase valued at 40 kcal of ME/kg (CD0), CD0 + β-mannanase (0.3 g/kg valued at 30 kcal of ME/kg) (CD70), CD0 + β-mannanase (0.3 g/kg valued at 45 kcal of ME/kg) (CD85), and CD0 + β-mannanase (0.3 g/kg valued at 60 kcal of ME/kg) (CD100), with 10 pen replicates. Pigs fed CD0 diet showed (*P* = 0.002) greater ADFI. However, pigs fed CD0 diet showed (*P* = 0.009) lower G:F than those provided CD70 or CD85 diets. A greater (*P* < 0.001) superoxide dismutase concentration was observed in pigs fed CD70 diet. Pigs fed CD85 diet showed (*P* = 0.002) greater digestible protein than pigs fed CD0 or CD100 diets. Pigs fed CD70 diet showed an increase of 11.3% in digestible protein than those fed CD0 diet. In addition, greater (*P* < 0.001) digestible energy was observed in pigs fed CD85 diet. Pigs fed CD0 or CD100 diets showed greater (*P* < 0.05) Firmicutes:Bacteroidota ratio than those fed CD85 diet. The Muribaculaceae was more abundant (*P* = 0.030) in pigs fed CD70 diet than in those fed CD0 diet. The *Prevotella* was more abundant (*P* = 0.045) in pigs fed CD85 diet than in those fed CD100 diet. In conclusion, β-mannanase supplementation in diets containing xylanase-phytase allows reducing 85 kcal of ME/kg because it improves gain to feed ratio, energy and protein usage, and backfat thickness without metabolic and intestinal ecosystem disorders in finisher pigs.

## 1. Introduction

Plant-based ingredients widely used in the diets of pigs possess significant amounts of antinutritional factors (1, 2). These antinutritional substances, such as β-mannans (1, 3–5), phytate molecules (3, 6), and xylans (4, 6), are not digested by endogenous enzymes, and compromise the use of nutrients and energy metabolism in non-ruminant animals (5).

Based on this, dietary supplementation of β-mannanase has been attributed to the hydrolysis of β-mannans reducing the immune response capacity induced by feeding (2), and energy expenditure for immune system activation (5). This nutritional strategy also allows the use of phytase enzyme, known to improve the availability of phosphorus and calcium in diets containing phytate molecules (3), and providing additional energy and improving energy efficiency (7). In addition, the antinutritional effects of non-starch polysaccharides (NSP) provided by xylans highlight the importance of using the xylanase enzyme (1). Xylanase breakdowns the plant cell wall releasing nutrients within the cell and reduce digesta viscosity (6).

Diets supplemented with a blend of these enzymes may be of economic-environmental-nutritional interest. Indeed, β-mannanase has been previously reported to reduce feed to gain ratio and increase nutrient ATTD (5). Greater phosphorus and lower neutral detergent fiber digestibility were reported when combined xylanase-phytase were supplemented in the diet of grower pigs (6). Greater blood glucose concentration and lower backfat thickness were observed in finisher pigs fed β-mannanase-xylanase (8); however, no effect on ATTD in grower pigs provided diets containing phytase and β-mannanase were observed (3).

To date, no studies have been conducted to assess the effects of the dietary association of these enzymes on the fecal microbiome, total digesta passage rate, and fecal consistency score in finisher pigs. Here, a study was conducted based on the hypothesis that β-mannanase supplementation in ME-reduced diets improves ATTD, intestinal digesta viscosity, and intestinal microbiome, supporting growth performance and health compared to the diet without β-mannanase supplementation.

Therefore, this study assessed the effects of β-mannanase associated with xylanase-phytase on growth performance, fecal score, biochemical and immunological blood profile, ATTD, total digesta passage rate, fecal microbiome, carcass traits, and meat quality in finisher pigs fed ME-reduced diets.

## 2. Materials and methods

### 2.1. Animals, experimental design, housing, and dietary treatments

A total of 40 entire male hybrid pigs (26.0 ± 0.9 kg BW) from a commercial line (Landrace × Large White) were used. Pigs were allotted to 1 of 4 dietary treatments in a randomized complete block design with 10 pen replicates and 1 animal per pen as the experimental unit. Blocks were based on the initial BW of pigs.

At the beginning of the experiment, animals were weighed and identified with numbered ear tags. Pigs were housed in a masonry facility with 2 rows (with a central aisle) of concrete floor pens (6.3 m^2^). All pens were equipped with a semiautomatic front feeder and a nipple waterer.

Room temperature and relative humidity were recorded by a data logger (Hygro-Thermometer, model RT811) located in the middle of the experimental facility. Temperature and ventilation were controlled via side curtains and trees on both sides of the facility. Room temperature and relative humidity averaged 20.4 ± 6.6°C and 63.6 ± 19.3%, respectively.

The experimental period lasted 52 days and was divided into 2 phases: finisher I (d 0 to 22) and finisher II (d 22 to 52). Diets (Table 1) were formulated to meet the nutritional requirements of pigs in each phase (9) and offered as mash, and *ad libitum*. All diets were corn- and soybean meal-based with industrial amino acids, and were isonutritional with variations only in soybean oil and inert content.

**Table 1:** Composition of diets provided to finisher pigs (as-fed basis).

**Item**	**Finisher I**				**Finisher II**			
	**CD0**	**CD70**	**CD85**	**CD100**	**CD0**	**CD70**	**CD85**	**CD100**
**Ingredients (%)**								
Ground corn, 7.86%	79.35	79.35	79.35	79.35	90.66	90.66	90.66	90.66
Soybean meal, 45.4%	16.23	16.23	16.23	16.23	4.76	4.76	4.76	4.76
Dicalcium phosphate	1.23	1.23	1.23	1.23	0.97	0.97	0.97	0.97
Limestone	0.52	0.52	0.52	0.52	0.45	0.45	0.45	0.45
Inert (kaolin)	-	0.33	0.51	0.69	0.59	0.92	1.10	1.28
Soybean oil	1.03	0.67	0.49	1.03	0.72	0.36	0.19	-
Sodium chloride	0.38	0.38	0.38	0.38	0.37	0.37	0.37	0.37
Premix^1^	0.30	0.30	0.30	0.30	0.30	0.30	0.30	0.30
Lysine sulfate, 54.6%	0.57	0.57	0.57	0.57	0.71	0.71	0.71	0.71
DL-methionine, 99.5%	0.12	0.12	0.12	0.12	0.10	0.10	0.10	0.10
L-threonine, 96.8%	0.15	0.15	0.15	0.15	0.18	0.18	0.18	0.18
L-tryptophan, 99%	0.03	0.03	0.03	0.03	0.05	0.05	0.05	0.05
L-valine, 95.5%	0.02	0.02	0.02	0.02	0.08	0.08	0.08	0.08
β-mannanase	-	0.03	0.03	0.03	-	0.03	0.03	0.03
Enramycin^2^	0.006	0.006	0.006	0.006	0.006	0.006	0.006	0.006
**Calculated composition**								
Metabolizable energy, kcal/kg	3,310	3,280	3,265	3,250	3,310	3,280	3,265	3,250
Crude protein, %	14.20	14.20	14.20	14.20	9.95	9.95	9.95	9.95
Lysine SID^3^, %	0.89	0.89	0.89	0.89	0.69	0.69	0.69	0.69
Methionine + cysteine SID, %	0.53	0.53	0.53	0.53	0.41	0.41	0.41	0.41
Threonine SID, %	0.58	0.58	0.58	0.58	0.45	0.45	0.45	0.45
Tryptophan SID, %	0.18	0.18	0.18	0.18	0.13	0.13	0.13	0.13
Valine SID, %	0.62	0.62	0.62	0.62	0.48	0.48	0.48	0.48
Total calcium, %	0.57	0.57	0.57	0.57	0.44	0.44	0.44	0.44
STTD phosphorus^4^, %	0.28	0.28	0.28	0.28	0.21	0.21	0.21	0.21
Total sodium, %	0.16	0.16	0.16	0.16	0.15	0.15	0.15	0.15

^1^Content per kg of premix: Mn sulfate, 5,400 mg/kg; Zn oxide, 13.50 g/kg; Fe sulfate, 10.50 g/kg; Cu sulfate, 2,100 mg/kg; I, 150 mg/kg; vitamin A, 900,000 IU/kg; vitamin D_3_, 180,000 IU/kg; vitamin E, 3,000 IU/kg; vitamin K_3_, 270 mg/kg; vitamin B_1_, 120 mg/kg; vitamin B_2_, 570 mg/kg; vitamin B_6_, 120 mg/kg; vitamin B_12_, 2,100 mcg/kg; niacin, 3,000 mg/kg; pantothenic acid, 1,950 mg/kg; folic acid, 75 mg/kg; Se, 90 mg/kg; phytase, 166.66 U/g; xylanase, 333.33 U/g.

^2^Erassen 80^®^: equivalent to 5 ppm of enramycin.

^3^SID: standardized ileal digestible.

^4^STTD: standardized total tract digestible.

Dietary treatments were: (1) a control diet containing isolated phytase and xylanase valued at 40 kcal of ME/kg (CD0), (2) CD0 + β-mannanase (0.3 g/kg valued at 30 kcal of ME/kg) (CD70), (3) CD0 + β-mannanase (0.3 g/kg valued at 45 kcal of ME/kg) (CD85), and (4) CD0 + β-mannanase (0.3 g/kg valued at 60 kcal of ME/kg) (CD100).

### 2.2. Traits of the tested enzymes

Xylanase (Sunhy Biology Co., Ltd, Wuhan, HB, China; registration no. PR-08978 03462) was a product obtained from *Trichoderma longibrachiatum*. A U of xylanase is the amount of enzyme that releases 1 micromol of reducing sugar from a xylan solution (5 mg/mL) at 37°C and pH 5.5. Phytase (Sunhy Biology Co., Ltd, Wuhan, HB, China; registration no. PR 000267-4.000005) was a product from *Aspergillus niger* with the activity of 1,000 U/g of dry solid at 37°C and pH 5.5. β-mannanase (Elanco Animal Health, Inc., São Paulo, SP, Brazil; registration no. SP-59122 30011, Hemicell^TM^ HT) was obtained from *Paenibacillus lentus*. A U of β-mannanase is the amount of enzyme that releases 0.72 mcg of reducing sugars (equivalent to D-mannose) per min from goma locust (mannans concentration of 88%) at 40°C and pH 7.5.

### 2.3. Growth performance and fecal consistency score

Animals had free access to diets and water throughout the experiment. Offered diets and leftovers were recorded daily using a digital scale (model UL-50, DIGI-TRON, Curitiba, PR, Brazil) to determine the average daily feed intake (ADFI, g/day). Pigs were weighed at the beginning and end of each experimental phase using a 2 bars digital scale (model ULB-3000, IWM bivolt, Curitiba, PR, Brazil). Initial BW (IBW, kg), final BW (FBW, kg), average daily gain (ADG, g/day), and gain to feed ratio (G:F, g:g) were determined.

Fecal consistency score was assessed *via* partial feces collection at the end of finisher phases. Before feces collection, all pens (08:00) were cleaned and animals were monitored for a 12-h period. During this period, fecal samples were collected right after defecation, except for the lower part that was in contact with the floor. Feces were packed in plastic bags and kept in a thermal box (4°C) until the end of the collection period. Then, the samples were homogenized and 2 subsamples of 110 g each were weighed in a scale (model M4102, Bel engineering, Monza, Italy) and dried in a forced-air oven (Tecnalbrand, SF-325 NM model; Piracicaba, SP, Brazil) at 55°C for 72 h for dry matter determination (10). Values were tabulated and classified according to fecal consistency, following the adapted methodology (11).

### 2.4. Blood sampling and blood profile analysis

Animals fasted for 8 h at the end of the finisher II phase. Blood samples (≅10 mL) were withdrawn from the anterior cranial vena cava using 1.2 × 40 mm needles and 20 mL syringes. Samples were transferred to 1 of 3 tubes containing potassium fluoride, EDTA, or no anticoagulant. All tubes were previously identified, placed into a thermal box (4°C), and sent to the blood laboratory for further analysis. Plasma or serum was isolated from blood by centrifugation (Centrilab analog centrifuge, model 80-2B) at 3,000 *g* for 10 min. Then, ≅3 mL of plasma or serum were transferred to previously identified polyethylene tubes (Eppendorf-type) and stored at −20°C until analysis of urea (enzymatic-colorimetric method), glucose (enzymatic-colorimetric method), total cholesterol (enzymatic-colorimetric method), total protein (enzymatic-biuret method), and albumin (bromocresol green colorimetric) of 10 animals per treatment.

All analyses were performed in the blood laboratory of Unioeste *via* spectrophotometry with the aid of an analyzer (Bel SPECTRO S05) using commercial kits (Gold Analisa Diagnóstica—Belo Horizonte, MG, Brazil).

Globulin was calculated as the difference between total protein and plasma albumin. Blood samples from 8 animals per treatment were stored at −80°C and sent to a private laboratory (Curitiba, PR, Brazil) where serum concentrations of superoxide dismutase (SOD), glutathione S-transferase, and immunoglobulins M were determined via the immunoturbidimetry method.

### 2.5. Apparent total tract digestibility and total digesta passage rate

The insoluble acid ash marker (IAA, celite^®^) was added to the diets (10 g/kg diet) at the end of the finisher II phase to assess ATTD using partial feces collection (indirect method) (12). The diets containing the marker were homogenized in a vertical mixer for 10 min. These diets were fed to pigs for 3 days before feces collection. On the fourth day, partial feces collection was performed following the adapted methodology (13). The beginning and end of the diet supply and the feed intake per pen were recorded. Feces were collected for 12 h on the last day of the supply of the diets containing the marker. During collection, feces were packed in polyethylene plastic bags (previously identified) and kept in thermal boxes containing ice (4°C). After this period, the feces were stored at −18°C for further analysis.

Afterward, the samples were thawed and homogenized. Two subsamples (110 g each) were weighed in a scale (bel engineering, model M4102, Monza, Italy) and dried in a forced-air oven (Tecnalbrand, SF-325 NM model; Piracicaba, SP, Brazil) at 55°C for 72 h, according to the methodologies (10). Then, the samples were ground in a micro-powder grinding mill (R-TE-350; Tecnal Scientific Equipment, Piracicaba, SP, Brazil) and stored in plastic containers previously identified for laboratory analyses.

Insoluble acid ash marker was analyzed *via* hydrochloric acid (4N) digestion, following the adapted procedures (13). The chemical composition of diets and feces samples was determined according to the methodologies described (10). The gross energy in diets and feces samples was determined in a bomb calorimeter (IKA^®^, model C200, USA).

Based on the results of laboratory analyses, the recovery percentage of IAA and the ATTD coefficients of dry matter (ADCDM), organic matter (ADCOM), crude protein (ADCCP), and gross energy (ADCGE) were calculated. Digestible nutrients and energy were determined as a percentage of digestible dry matter (DDM), digestible organic matter (DOM), digestible protein (DP), and kcal/kg of digestible energy (DE), according to the established equations (12).

The total digesta passage rate was assessed *via* fecal marker excretion at the end of the finisher phases, according to the adapted methodology (14). Before supplying the diets containing the marker, a quantified portion of the diet was weighed with 1.5% of the marker (iron oxide) and homogenized to ensure the intake in a single meal. One h before the evaluation, all diet was removed from the feeder of pens and placed in identified containers to be returned to the respective feeder afterward. Diets containing the marker were supplied following the same sequence used to withdraw diets without marker. The supplying time and the time when animals consumed all the marked diet (h 0) were recorded per pen. Pens were monitored to identify the defection of marked feces. The defecation time was recorded accordingly. The total digesta passage rate was calculated based on the time (in min) between the marked diet consumption and the excretion of marked feces.

### 2.6. Fecal microbiome

At the end of finisher II phase, rectum feces samples from 6 pigs per treatment were collected and immediately placed in sterile Eppendorf-type tubes using swabs. Right after collection, the samples were stored at −80°C until analysis.

A commercial kit (ZR Fecal DNA MiniPrep^®^ from Zymo Research) was used to extract DNA from samples following the manufacturer's instructions. The integrity of the extracted DNA was assessed *via* 1% agarose gel electrophoresis. The extracted DNA was quantified *via* spectrophotometry at 260 nm.

A segment of approximately 460 bases of the hypervariable region V3-V4 of the ribosomal gene 16S rRNA was amplified using the universal primers described by the methodology. The PCR conditions were as follows: 95°C for 3 min, 25 cycles at 95°C for 30 s, 55°C for 30 s, and 72°C for 30 s, followed by a step at 72°C for 5 min. A metagenomics library was built from the amplified using a commercial kit (Nextera DNA Library Preparation Kit, Illumina^®^). The amplified were pooled and sequenced in Illumina's MiSeq^TM^ sequencer^®^ (15).

Readings were analyzed in the quantitative insights into microbial ecology (QIIME2) platform (16). The following procedures were performed: removal of low-quality sequences, filtration, chimera's removal, and taxonomic classification. Sequences were classified into bacterial genera *via* amplicon sequence variants (ASVs) identification, in this case, the homology between sequences when compared against a database. The 2019 edition (SILVA 138) of the SILVA ribosomal sequence database (17) was used to compare the sequences.

To generate the classification of bacterial communities *via* ASVs identification, 25,610 readings per sample were used. Thus, data were normalized and samples with different number of readings were not compared. The samples of identifiers 29,160 and 29,167 were removed due to the low number of readings (< 15,000). They were retrieved after the quality filtering steps.

### 2.7. Slaughter procedures, carcass traits, and meat quality

On day 52 of the experimental period, all animals (*n* = 10/treatment) were fasted for 12 h and then transported for 6 h (a total of 18 h of fasting) to a commercial abattoir (Medianeira, PR, Brazil) with federal certification. Pigs were slaughtered using carbon dioxide stunning, followed by exsanguination.

All analyses were performed and calculated according to the methodologies described (18). The quantitative carcass traits such as backfat thickness, muscle percentage in the carcass, lean meat percentage, and lean meat amount were measured in the slaughterhouse using a swine carcass typing pistol (model UltraFom 300, Carometec). The carcass weight was determined using a scale placed in the slaughter line. Then, hot carcass yield, meat yield, and amount of chilled meat were calculated.

Carcass length was measured after a cold shock in the cold chamber. Measurements were taken from the cranial edge of the atlas to the cranial edge of the aitch bone. A sample (≅30 cm) of the *l. thoracis* muscle was collected between the last thoracic vertebra and the first lumbar vertebra (caudal to cranial direction). Samples were immediately packed in the identified polyethylene plastic bags, placed in thermal boxes (4°C), and transported to the Animal Products Technology Laboratory (APTL) belonging to Unioeste.

Then, pH value in the *l. thoracis* muscle was measured using a portable pHmeter (model AK103, Asko produtos eletrônicos Ltda, São Leopoldo, RS, Brazil) in the area of the last rib 4 and 24 h *post mortem*. For the measurements taken 4 h *post mortem*, the carcasses were submitted to 180 min of cooler shock, as follows: first stage: from −18°C to −15°C; second stage: from −15°C to −12°C; and third stage: from −10°C to −8°C.

At the APTL, samples were refrigerated (≅2°C) for 24 h and then the backfat thickness and loin depth were measured using a digital pachymeter (MTX, stainless hardened). To determine the loin eye area (LEA) of the *l. thoracis* muscle, samples were scanned using a scanner printer (Officejet 4500 Desktop - G510a, HP, São Paulo, SP, Brazil). A black box was used to block the lighting and improve the image quality. Then, readings were performed using a Software (imageJ 1.53e - Java).

Meat color was assessed after muscle oxygenation via air exposure for 15 min. Color analyses were performed using a Minolta CR400 colorimeter device (Konica Minolta Holdings, inc. Tokyo, Japan) and the results were expressed using the CIELAB color system. Color parameters were measured as L^*^ (luminosity), a^*^ (red-green component), and b^*^ (yellow-blue component), which represent the saturation (chroma or purity) and the tint (color or hue). With these results, the saturation of the *l. thoracis* muscle was calculated.

Marbling was determined using photographic standards and a 7-point scale (1 = traces of marbling and, 7 = excessive marbling). The subjective color analysis was performed using a 6-point scale (1 = light color and, 6 = trend to red).

Afterward, samples were boned and the *l. thoracis* muscle was cross-sectioned into four 2.5-cm subsamples. The subsamples were used to determine drip loss (DL), thaw loss (TL), cooking loss (CL), shear force (SF), and chemical analyses. Subsample 1 was used to assess DL. The remaining subsamples were packed in the identified polyethylene bags and stored at−18°C until analyses. The losses were expressed as the percentage of lost water in relation to the original sample weight. Cooking loss was performed sequentially in a grill (Britannia brand, multi grill 2). Shear force analysis was performed using 6 cores (1.5 cm) removed from subsample 2 (TL and CL sequentially) using a stainless-steel cylinder sampler. Subsequently, the cores were submitted to a TA.HD.plus texture meter (model Texture Analyser, Stable Micro Systems) equipped with a standard shear blade calibrated for force (15 g), deformation (20 mm), and speed (2.0 mm/s).

Subsample 3 was thawed in a refrigerator at a controlled temperature (4°C). Fat and connective tissue were withdrawn using a knife. Then, the subsamples were ground in a microprocessor and packed in the originally identified bags to determine moisture, ash, and crude protein. The ether extract was performed according to the AOCS methodology (Am 5-04, 2017) using an Ankom extractor (model XT15, NY, USA). Subsample 4 was kept frozen as a backup. The *in vivo* loin depth and backfat thickness were assessed in the lumbar area P2 in finisher II pigs using an Aloka ultrasound (Echo Camera model - SSD-500 vet, Tokyo, Japan).

### 2.8. Statistical procedures

A Student standardized residuals analysis was performed before one-way analysis of covariance (ANCOVA) and variance (ANOVA), in which values >3 standard deviations were considered outliers. The normality of experimental errors and the homogeneity of variance of errors among treatments were evaluated using Shapiro-Wilk and Levene tests, respectively. For antioxidant enzyme data, outliers were identified via ROUT test (*Q* = 1%) and the normality was assessed *via* D'Agostino-Pearson test. Data on growth performance were analyzed using the following model:


$$\begin{array}{l} Y_{ijk}=\;\mu \;+\;T_{i}+\;b_{j}+\;\beta \;\left(X_{ijk}-\bar{X}...\right)\;+\;\varepsilon_{ijk} \end{array}$$

The effects of the factors in the model were described as: Y_ijk_ = average observation of the dependent variable in each plot, measured in the i-th class of treatment, in the j-th block, and the k-th replication; μ = overall mean effect; T_i_ = fixed effect of treatment classes, i = (1, 2, 3, and 4); b_j_ = random effect of block, j = (1 and 2); β = regression coefficient of Y over X; X_ijk_ = average observation of the covariate (initial BW) in each plot, measured in the i-th class of treatment, in the j-th block, and the k-th replication; ${\bar{X}}_{\ldots }$ = overall mean for covariate X; ε_ijk_ = random error of the plot associated with level i, block j, and replication k. For other variables, the statistical model used was the one mentioned above, no covariate effect.

Treatment effect on dependent variables was verified *via* ANCOVA or ANOVA. Treatment significance was set at *P* < 0.10 when the power of the test was < 80%. Multiple comparisons among treatment means were performed according to the *post hoc* test of Tukey and t-Student at 5% and 10% of probability, respectively. All statistical analyses were performed using the procedures of the SAS University Edition (SAS Inst. Inc., Cary, NC, USA). All normally distributed data were reported as means and their pooled SEM.

For the fecal microbiome, the statistical comparison among the groups in the analyses of alpha diversity and the relative abundances of taxa among all experimental groups was performed *via* Wilcoxon non-parametric test at *P* < 0.05. Statistical analyses for beta diversity were performed through permutational multivariate analysis of variance (PERMANOVA) in the QIIME2 pipeline. A total of 10,000 permutations was used. Alpha diversity analyses were calculated using phyloseq (19) and microbiome (20) libraries.

## 3. Results

### 3.1. Growth performance and fecal consistency score

Pigs fed CD0 diet showed (*P* = 0.002) greater ADFI than pigs fed other dietary treatments (Table 2). However, pigs fed CD0 diet showed (*P* = 0.009) lower G:F than those provided CD70 or CD85 diets. Although no difference among dietary treatments was observed in the finisher II phase, pigs fed ME-reduced diets containing the enzymes combination-maintained growth performance. No dietary treatment effect on the fecal consistency score was observed in finisher pigs.

**Table 2:** Effect of β-mannanase supplementation in diets containing xylanase-phytase and reduced metabolizable energy on performance and fecal consistency score in finisher pigs^1^.

**Item^2^**	**Treatments** ^**3**^				**SEM^4^**	***P-*value**
	**CD0**	**CD70**	**CD85**	**CD100**		
**Finisher I (d 0 to 22)**						
FBW, kg	100.90	96.45	98.10	97.95	0.90	0.505
ADFI, g	3,076^a^	2,590^b^	2,598^b^	2,753^b^	0.04	0.002
ADG, g	1,291	1,232	1,225	1,249	0.01	0.525
G:F, g:g	0.42^b^	0.47^a^	0.46^a^	0.45^ab^	0.66	0.009
FCS	1.10	1.00	0.90	0.90	0.05	0.557
**Finisher II (d 22 to 52)**						
FBW, kg	138.30	133.65	133.45	134.50	1.36	0.518
ADFI, g	3,214	3,165	2,972	3,251	0.08	0.637
ADG, g	1,244	1,241	1,178	1,219	0.05	0.962
G:F, g:g	0.38	0.39	0.39	0.37	1.38	0.922
FCS	1.10	0.79	0.80	0.78	0.10	0.614
**Overall period (d 0 to 52)**						
ADFI, g	3,155	2,923	2,815	3,040	0.05	0.141
ADG, g	1,264	1,237	1,195	1,231	0.03	0.467
G:F, g:g	0.40	0.42	0.42	0.40	0.00	0.418

^a, b^Means followed by different lowercase letters on the same row differ by Tukey *post hoc* test (P < 0.05).

^1^Data are means of 10 pens replicates per treatment and 1 pig per pen as an experimental unit.

^2^IBW: initial body weight, FBW: final body weight, ADFI: average daily feed intake, ADG: average daily gain, G:F: gain to feed ratio, FCS: fecal consistency score (score 0: < 70% normal feces); score 1: 70–75% soft feces, score 2: 75–80% semi-solid feces, and score 3: > 80% liquid feces.

^3^(1) control diet containing isolated phytase and xylanase valued at 40 kcal of ME/kg (CD0), (2) CD0 + β-mannanase (0.3 g/kg valued at 30 kcal of ME/kg) (CD70), (3) CD0 + β-mannanase (0.3 g/kg valued at 45 kcal of ME/kg) (CD85), and (4) CD0 + β-mannanase (0.3 g/kg valued at 60 kcal of ME/kg) (CD100).

^4^SEM: pooled standard error of the mean.

### 3.2. Blood biochemical and immune profile

A greater (*P* < 0.001) SOD concentration was observed in pigs fed CD70 diet compared to other dietary treatments (Table 3). No dietary treatment effect on the biochemical blood profile was observed in finisher pigs.

**Table 3:** Effect of β-mannanase supplementation in diets containing xylanase-phytase and reduced metabolizable energy on blood biochemical and immunological profile in finisher pigs on day 52^1^.

**Item^2^**	**Treatments** ^**3**^				**SEM^4^**	***P-*value**
	**CD0**	**CD70**	**CD85**	**CD100**		
Albumin, g/dL	3.57	3.57	3.55	3.84	0.06	0.337
Total cholesterol, mg/dL	86.86	98.60	92.31	92.14	2.52	0.485
Glucose, mg/dL	77.45	73.09	69.45	68.86	1.80	0.303
Urea, mg/dL	18.42	21.05	17.01	19.54	0.91	0.482
Total protein, g/dL	6.12	5.79	6.34	6.04	0.13	0.569
Globulin, g/dL	2.54	2.22	2.79	2.20	0.13	0.365
GST, μmol/min/mg protein	5.57	5.98	6.52	6.98	0.32	0.467
SOD, U/mg protein	219.70^b^	279.10^a^	216.90^b^	221.40^b^	5.94	< 0.001
IgM, mg/dL	83.01	99.41	95.24	69.53	5.09	0.153

^a, b^Means followed by different lowercase letters on the same row differ by Tukey *post hoc* test (P < 0.05).

^1^Biochemical profile data are means of 10 pigs per treatment.

^2^GST: glutathione S-transferase, SOD: superoxide dismutase, and IgM: immunoglobulin M (8 pigs per treatment).

^3^(1) control diet containing isolated phytase and xylanase valued at 40 kcal of ME/kg (CD0), (2) CD0 + β-mannanase (0.3 g/kg valued at 30 kcal of ME/kg) (CD70), (3) CD0 + β-mannanase (0.3 g/kg valued at 45 kcal of ME/kg) (CD85), and (4) CD0 + β-mannanase (0.3 g/kg valued at 60 kcal of ME/kg) (CD100).

^4^SEM: pooled standard error of the mean.

### 3.3. Apparent total tract digestibility and total digesta passage rate

Pigs fed CD85 diet showed (*P* = 0.002) greater DP than pigs fed CD0 or CD100 diets. Pigs fed CD70 diet showed an increase of 11.3% in DP than those fed CD0 diet (Table 4). In addition, greater (*P* < 0.001) DE was observed in pigs fed CD85 diet compared to other dietary treatments. No effect of dietary treatments on the passage rate of total digesta was observed in finisher pigs.

**Table 4:** Effect of β-mannanase supplementation in diets containing xylanase-phytase and reduced metabolizable energy on apparent total tract digestibility (dry matter basis) on day 52, and total digesta passage rate in finisher pigs on days 22 and 52^1^.

**Item^2^**	**Treatments** ^**3**^				**SEM^4^**	***P-*value**
	**CD0**	**CD70**	**CD85**	**CD100**		
ADCDM (%)	83.50	84.09	84.01	83.02	0.30	0.598
ADCCP (%)	74.36	76.89	78.14	75.94	0.76	0.341
ADCOM (%)	86.09	86.91	87.03	86.08	0.31	0.576
ADCGE (%)	82.14	83.89	84.54	83.47	0.41	0.187
DDM (%)	82.75	83.27	83.06	81.95	0.32	0.509
DP (%)	8.61^c^	9.59^ab^	9.71^a^	8.99^bc^	0.11	0.002
DOM (%)	82.43	82.89	82.68	81.30	0.30	0.279
DE (kcal/kg)	3,697^b^	3,805^b^	4,035^a^	3,813^b^	27.07	< 0.001
TDP_I_ on day 22 (min)	1,656	1,604	1,571	1,475	36.30	0.357
TDP_II_ on day 52 (min)	2,179	2,409	2,414	2,353	117.12	0.895

^a, b, c^Means followed by different lowercase letters on the same row differ by Tukey *post hoc* test (P < 0.05).

^1^Data are means of 10 pigs per treatment.

^2^Apparent digestibility coefficient of dry matter (ADCDM), organic matter (ADCOM), protein (ADCCP), gross energy (ADCGE); digestible nutrients: digestible dry matter (DDM), digestible organic matter (DOM), digestible protein (DP); digestible energy (DE); TDP: total digesta passage rate in finisher I (TDPI) and II (TDPII).

^3^(1) control diet containing isolated phytase and xylanase valued at 40 kcal of ME/kg (CD0), (2) CD0 + β-mannanase (0.3 g/kg valued at 30 kcal of ME/kg) (CD70), (3) CD0 + β-mannanase (0.3 g/kg valued at 45 kcal of ME/kg) (CD85), and (4) CD0 + β-mannanase (0.3 g/kg valued at 60 kcal of ME/kg) (CD100).

^4^SEM: pooled standard error of the mean.

### 3.4. Fecal microbiome

**Figure 1:**
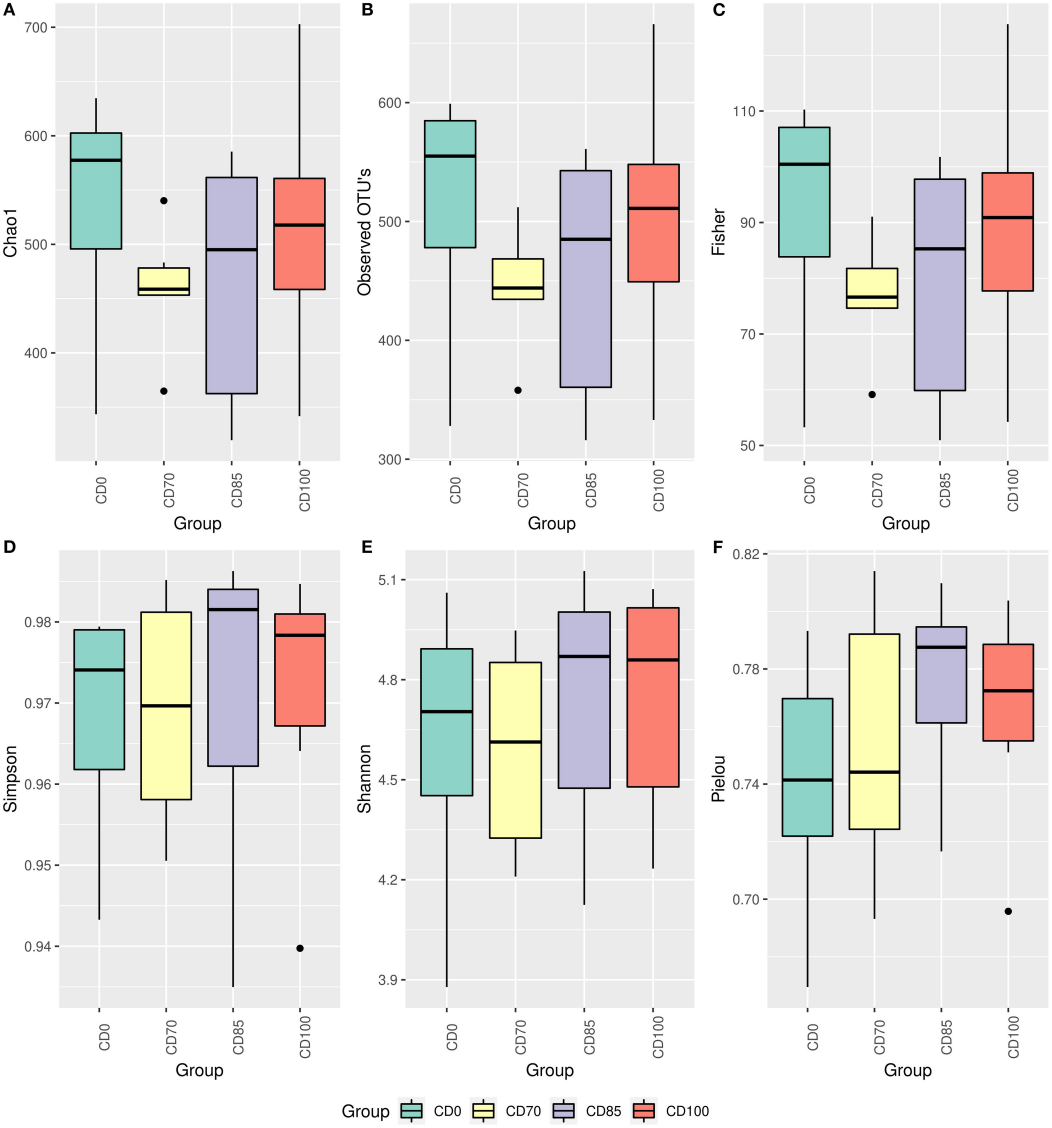
Alpha diversity estimated by parameters Chao1 **(A)**, observed OTUs **(B)**, Fisher **(C)**, Simpson **(D)**, Shannon **(E)**, and Pielou **(F)** in finisher pigs fed 1 of 4 dietary treatments: a control diet containing isolated phytase and xylanase valued at 40 kcal of ME/kg (CD0), CD0 + β-mannanase (0.3 g/kg valued at 30 kcal of ME/kg) (CD70), CD0 + β-mannanase (0.3 g/kg valued at 45 kcal of ME/kg) (CD85), and CD0 + β-mannanase (0.3 g/kg valued at 60 kcal of ME/kg) (CD100). Data are averages of 6 pigs per dietary treatment.

**Figure 2:**
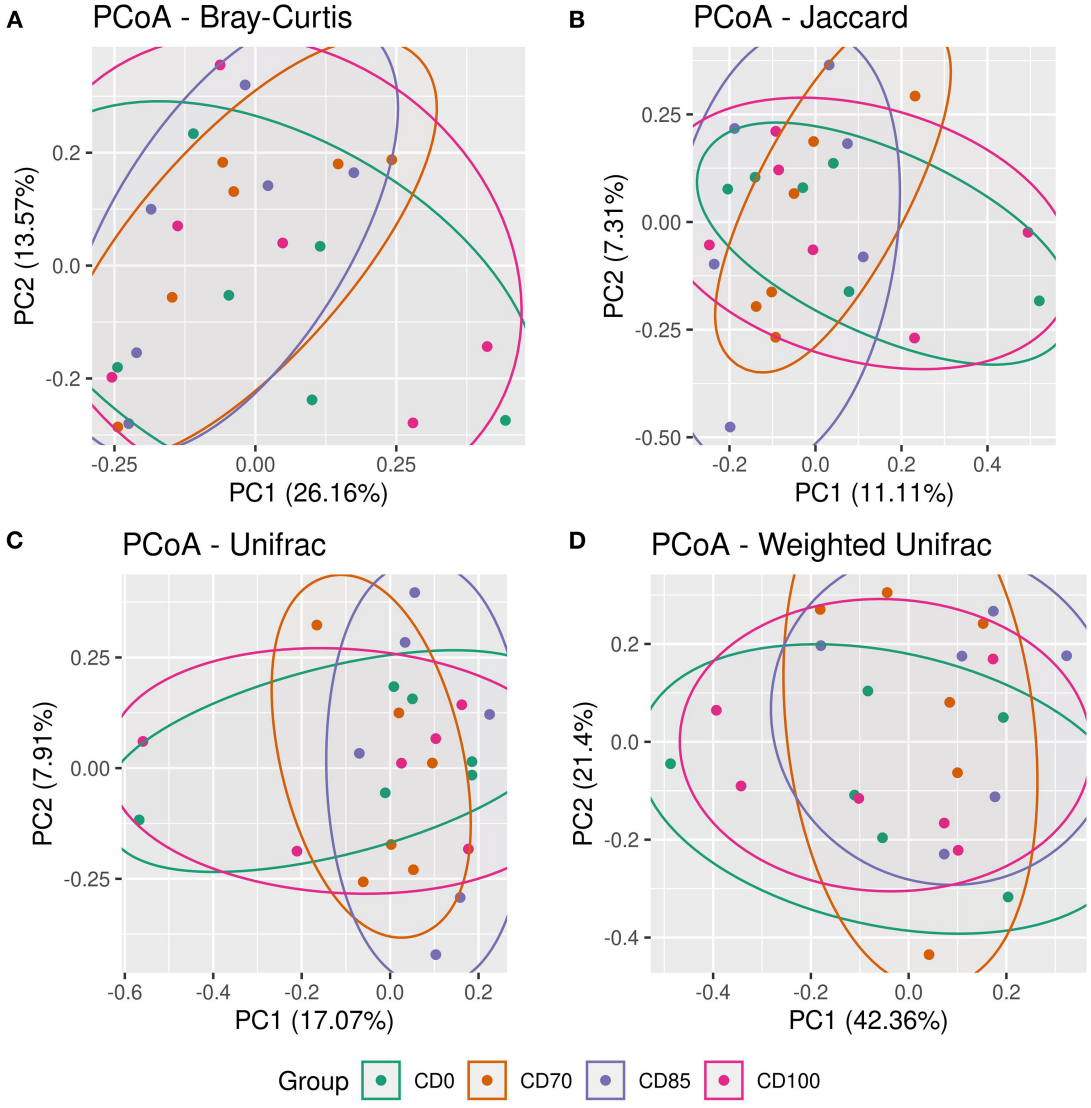
Beta diversity estimated by parameters Bray-Curtis **(A)**, Jaccard **(B)**, Unifrac **(C)**, and Weighted Unifrac **(D)** in finisher pigs fed 1 of 4 dietary treatments: a control diet containing isolated phytase and xylanase valued at 40 kcal of ME/kg (CD0), CD0 + β-mannanase (0.3 g/kg valued at 30 kcal of ME/kg) (CD70), CD0 + β-mannanase (0.3 g/kg valued at 45 kcal of ME/kg) (CD85), and CD0 + β-mannanase (0.3 g/kg valued at 60 kcal of ME/kg) (CD100). Data are averages of 6 pigs per dietary treatment.

No difference among treatments was observed *via* the alpha diversity test (Shannon, Evenness Pielou, Simpson Index, Fisher, total number of observed OTUs, and Chao 1) in finisher pigs (Figure 1). Beta diversity was estimated *via* Bray-Curtis, Jaccard, UniFrac, and Weighted Unifrac parameters (Figure 2); however, no differences among dietary treatments were observed in finisher pigs.

**Figure 3:**
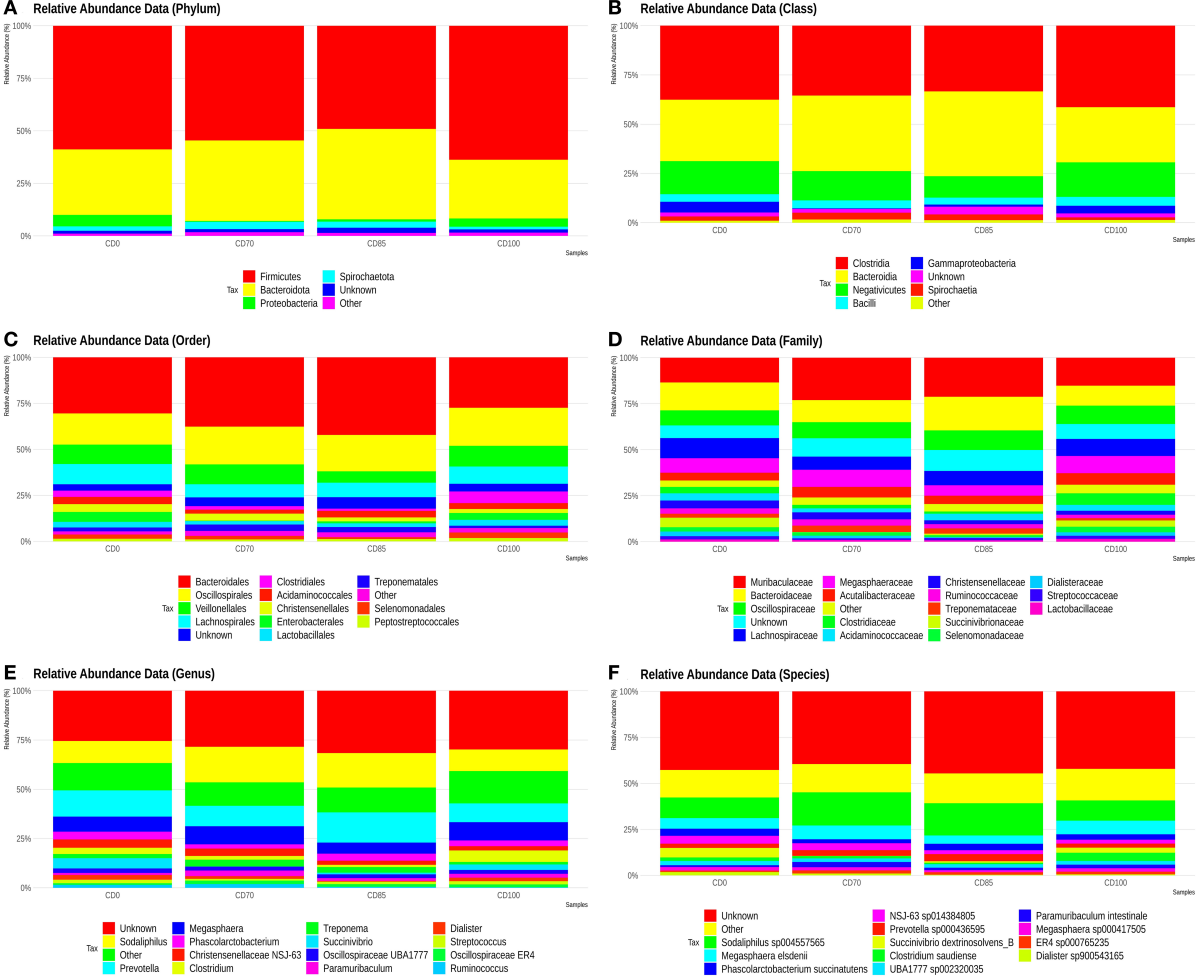
Relative abundance of phyla **(A)**, classes **(B)**, orders **(C)**, families **(D)**, genera **(E)**, and species **(F)** presents in finisher pigs fed 1 of 4 dietary treatments: a control diet containing isolated phytase and xylanase valued at 40 kcal of ME/kg (CD0), CD0 + β-mannanase (0.3 g/kg valued at 30 kcal of ME/kg) (CD70), CD0 + β-mannanase (0.3 g/kg valued at 45 kcal of ME/kg) (CD85), and CD0 + β-mannanase (0.3 g/kg valued at 60 kcal of ME/kg) (CD100). Data are averages of 6 pigs per dietary treatment.

The most abundant phyla we observed were Firmicutes, Bacteroidota (previously described as Bacteroidetes), Proteobacteria, and Spirochaetota (previously described as Spirochaetes) (Figure 3A). The classes Clostridia, Bacteroidia, Negativicutes, Bacilli, Gammaproteobacteria, and Spirochaetia showed the largest populations (Figure 3B). The most abundant orders were Bacteroidales, Oscillospirales, Veillonellales, Lachnospirales, Clostridiales, Acidaminococcales, Christensenellales, Enterobacterales, Lactobacillales, Treponematales, Selenomonadales, and Peptostreptococcales (Figure 3C).

The most abundant families were Muribaculaceae, Bacteroidaceae, Oscillospiraceae, Lachnospiraceae, Megasphaeraceae, Acutalibacteraceae, Clostridiaceae, Acidaminococcaceae, Christensenellaceae, Ruminococcaceae, Treponemataceae, Succinivibriononaceae, Selenomonadaceae, Dialisteraceae, Streptococcaceaeaceae, and Lactobacillaceae (Figure 3D).

The most abundant genera were *Sodaliphilus, Prevotella, Megasphaera, Phascolarctobacterium, Christensenellaceae NSJ-63, Clostridium, Treponema, Succinivibrio, Oscillospiraceae UBA1777, Paramuribaculum, Dialister, Streptococcus, Oscillospiraceae ER4*, and *Ruminococcus* (Figure 3E).

The species *Sodaliphilus sp004557565, Megasphaera elsdenii, Phascolarctobacterium succinatutens, NSJ-63 sp014384805, Prevotella sp000436595, Succinivibrio dextrinosolvens_B, Clostridium saudiense, UBA1777 sp002320035, Paramuribaculum intestinale, Megasphaera sp000417505, ER4 sp000765235*, and *Dialister sp900543165* showed the largest abundances (Figure 3F).

**Figure 4:**
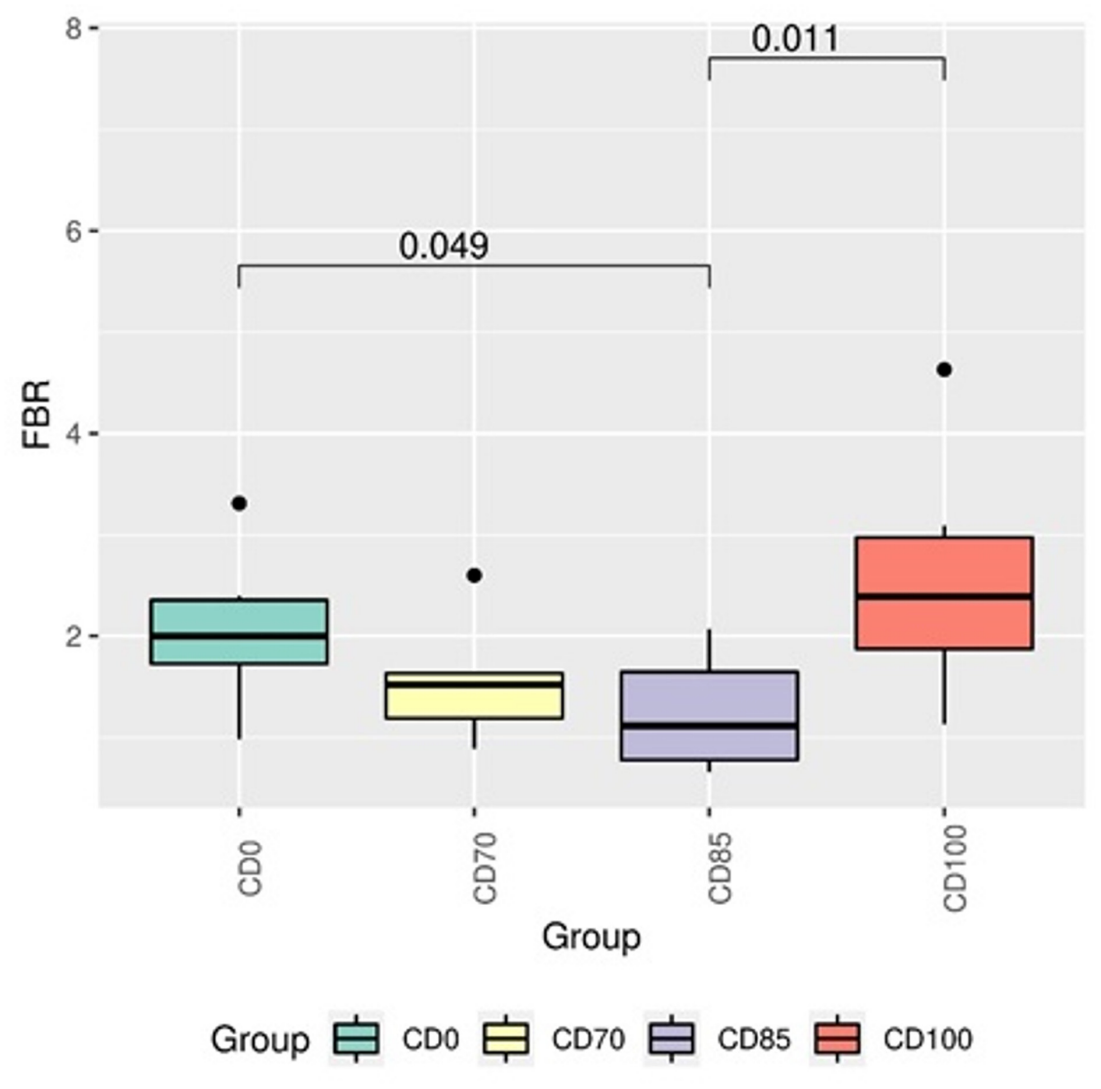
Firmicutes:Bacteroidetes ratio in finisher pigs fed 1 of 4 dietary treatments: a control diet containing isolated phytase and xylanase valued at 40 kcal of ME/kg (CD0), CD0 + β-mannanase (0.3 g/kg valued at 30 kcal of ME/kg) (CD70), CD0 + β-mannanase (0.3 g/kg valued at 45 kcal of ME/kg) (CD85), and CD0 + β-mannanase (0.3 g/kg valued at 60 kcal of ME/kg) (CD100). Data are averages of 6 pigs per dietary treatment. Means differed by Wilcoxon test (*P* < 0.05).

**Figure 5:**
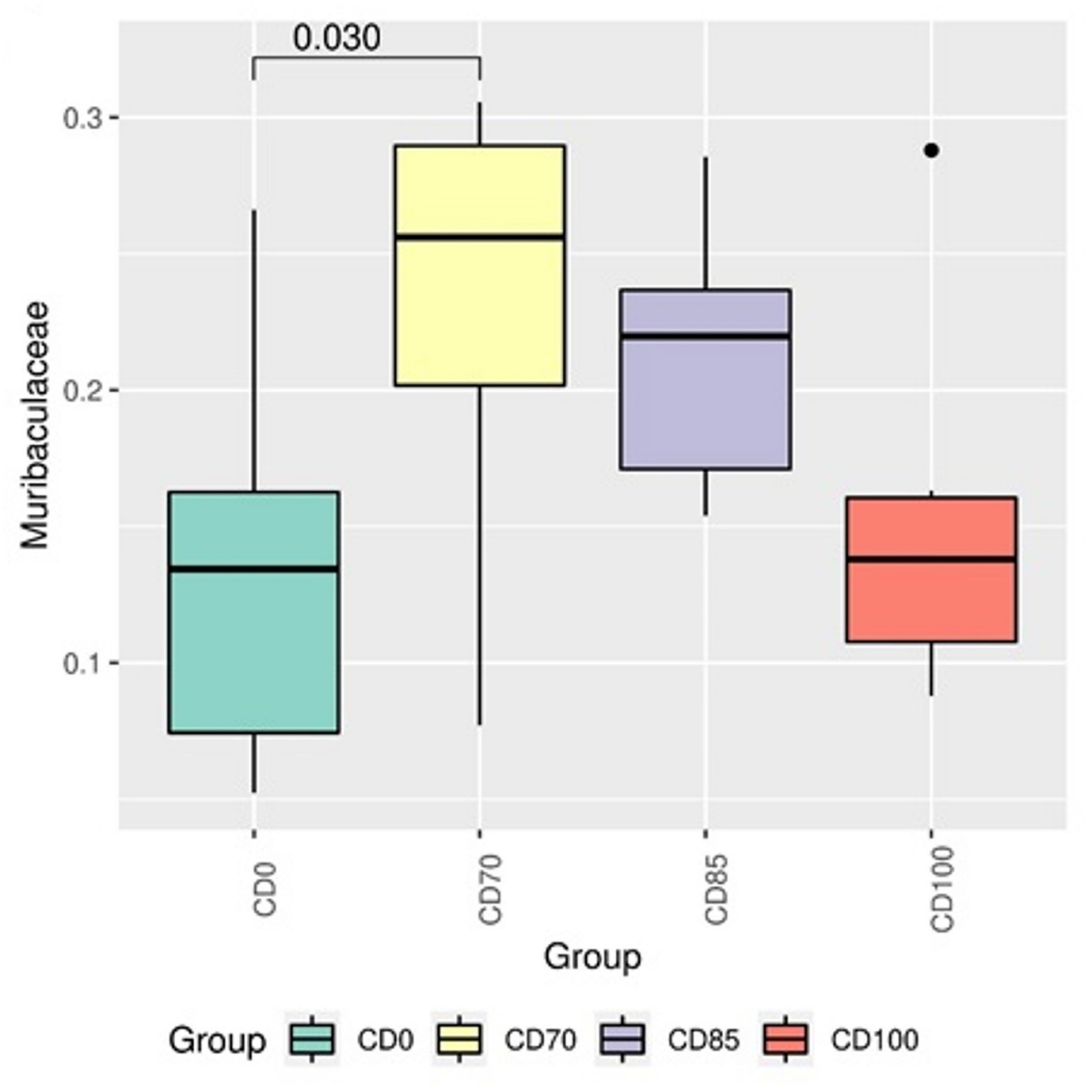
Differential abundance analysis of taxon of the Muribaculaceae family in finisher pigs fed 1 of 4 dietary treatments: a control diet containing isolated phytase and xylanase valued at 40 kcal of ME/kg (CD0), CD0 + β-mannanase (0.3 g/kg valued at 30 kcal of ME/kg) (CD70), CD0 + β-mannanase (0.3 g/kg valued at 45 kcal of ME/kg) (CD85), and CD0 + β-mannanase (0.3 g/kg valued at 60 kcal of ME/kg) (CD100). Data are averages of 6 pigs per dietary treatment. Means differed by Wilcoxon test (*P* < 0.05).

**Figure 6:**
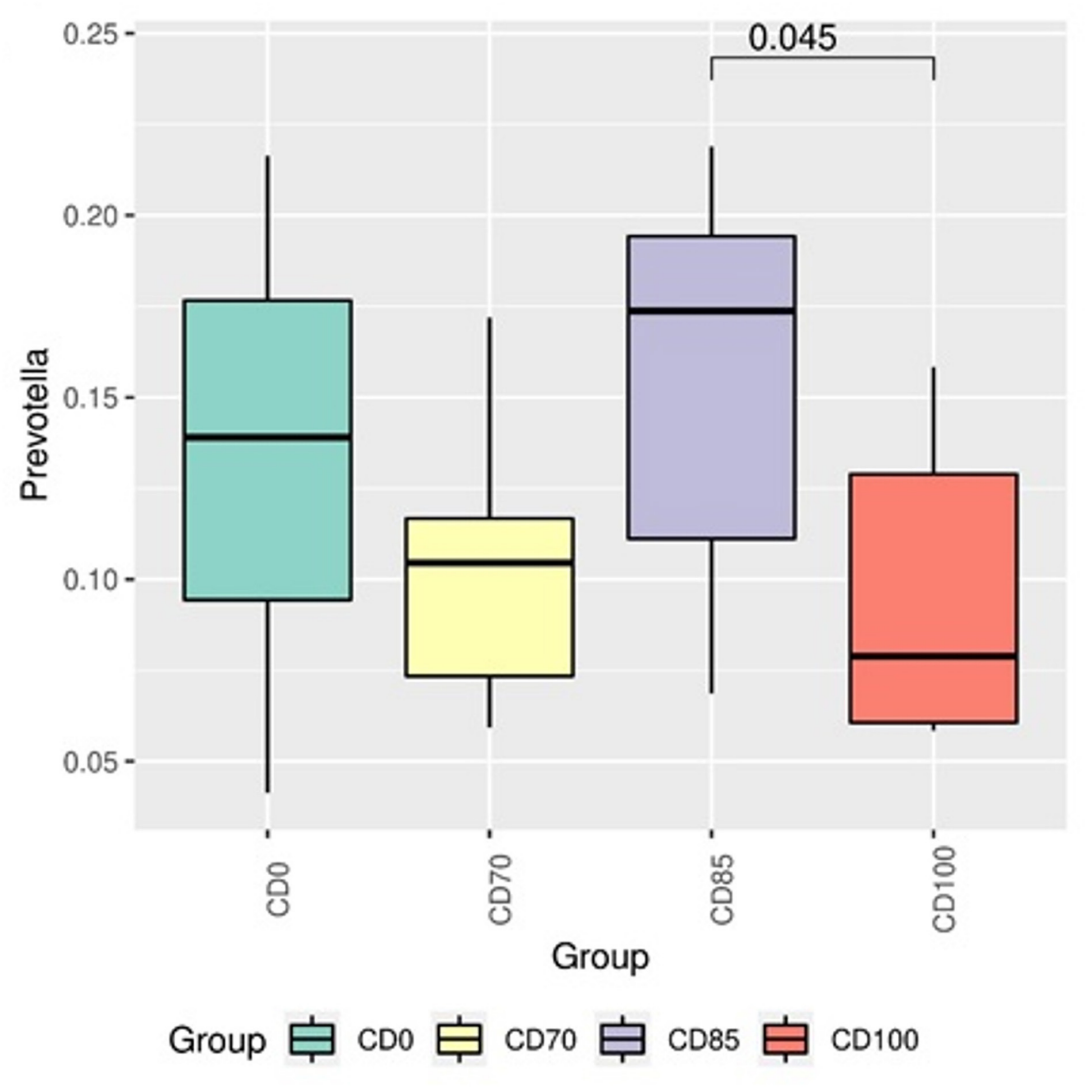
Differential abundance analysis of taxon of the *Prevotella* genus in finisher pigs fed 1 of 4 dietary treatments: a control diet containing isolated phytase and xylanase valued at 40 kcal of ME/kg (CD0), CD0 + β-mannanase (0.3 g/kg valued at 30 kcal of ME/kg) (CD70), CD0 + β-mannanase (0.3 g/kg valued at 45 kcal of ME/kg) (CD85), and CD0 + β-mannanase (0.3 g/kg valued at 60 kcal of ME/kg) (CD100). Data are averages of 6 pigs per dietary treatment. Means differed by Wilcoxon test (*P* < 0.05).

In addition, pigs fed CD0 diet showed (*P* = 0.049) greater Firmicutes:Bacteroidota ratio (FBR) than those provided with CD85 diet (Figure 4). However, pigs fed CD100 diet showed (*P* = 0.011) greater FBR than those fed CD85 diet. We analyzed only the taxon that showed different (*P* < 0.05) average relative abundance among dietary treatments. Therefore, the Muribaculaceae family was more abundant (*P* = 0.030) in pigs fed CD70 diet than in those fed CD0 diet (Figure 5). In addition, the *Prevotella* genus was more abundant (*P* = 0.045) in pigs fed CD85 diet than in those fed CD100 diet (Figure 6).

### 3.5. Carcass traits and meat quality

Pigs fed CD0 diet showed (*P* = 0.094) greater backfat thickness measured with ultrasound than pigs fed CD70 or CD85 diets (Table 5). In addition, animals fed CD0 or CD85 diets showed (*P* = 0.060) greater pH_24h_ in the *l. thoracis* muscle than those fed CD100 diet.

**Table 5:** Effect of β-mannanase supplementation in diets containing xylanase-phytase and reduced metabolizable energy on carcass traits and meat quality in finisher pigs on day 52^1^.

**Item^2^**	**Treatments** ^**3**^				**SEM^4^**	***P-*value**
	**CD0**	**CD70**	**CD85**	**CD100**		
**Quantitative traits**						
CL (cm)	96.05	98.77	100.66	101.40	1.27	0.466
HCW (kg)	96.70	94.72	91.68	93.92	1.06	0.447
Musc (%)	56.77	54.62	56.77	55.90	1.08	0.903
HCY (kg)	69.23	70.74	69.28	69.87	0.56	0.785
LM (%)	56.63	57.03	57.14	57.01	0.48	0.986
LM (kg)	54.93	54.32	52.35	53.44	0.76	0.677
CM (%)	51.59	53.34	50.72	51.20	0.68	0.588
MY (%)	53.90	56.14	57.01	55.19	0.58	0.271
LEA (cm^2^)	60.23	54.12	59.77	57.90	1.18	0.263
LDpaq (mm)	63.40	63.04	61.88	63.31	0.88	0.932
LDult (mm)	57.00	55.00	55.30	55.90	0.59	0.676
BFTpaq (mm)	21.44	20.04	17.58	19.20	0.71	0.284
BFTpis (mm)	18.62	17.67	17.38	18.00	0.79	0.958
BFTult (mm)	17.90^a^	15.33^b^	15.20^b^	16.90^ab^	0.04	0.094
**Qualitative traits**						
pH_4h_	6.23	6.15	6.11	6.08	0.04	0.631
pH_24h_	6.14^a^	5.75^ab^	5.95^a^	5.32^b^	0.11	0.060
DL (%)	7.03	6.34	7.98	7.06	0.27	0.214
TL (%)	9.49	8.92	9.43	8.62	0.35	0.800
CL (%)	28.27	25.37	26.10	29.05	0.75	0.262
SF (kgf/seg)	4,138	3,493	3,909	4,367	142.97	0.159
L^*^	45.33	46.09	46.39	45.33	0.29	0.481
a^*^	5.59	5.67	5.25	5.26	0.13	0.571
b^*^	3.07	3.57	3.13	2.84	0.10	0.107
Chroma	6.38	6.72	6.12	5.99	0.15	0.387
Color score	3.61	3.05	2.83	3.45	0.13	0.137
Marbling degree	3.33	3.00	3.22	3.20	0.15	0.907
Ash (%)	1.19	1.18	1.21	1.20	0.01	0.884
Crude protein (%)	24.54	25.01	24.25	24.47	0.23	0.716
Ether extract (%)	3.31	3.45	2.92	3.60	0.21	0.736
Moisture (%)	73.55	73.13	73.47	73.55	0.16	0.777

^a, b^Means followed by different lowercase letters on the same row differ by *post hoc* t-Student test (P < 0.10).

^1^Data are means of 10 pigs per treatment.

^2^CL: carcass length, HCW: hot carcass weight, Musc: muscle percentage in the carcass, HCY: hot carcass yield, LM (%): lean meat percentage, LM (kg): lean meat amount, CM: chilled meat, MY: meat yield, LEA: loin eye muscle area, LDpaq: loin depth measured using a pachymeter, LDult: loin depth measured *in vivo* using ultrasound at the end of finisher II phase, BFTpaq: backfat thickness measured using a pachymeter, BFTpis: backfat thickness measured using a Hennessy pistol, BFTult: backfat thickness measured *in vivo* using ultrasound at the end of finisher II phase, pH_4h_: pH at 4 h after slaughter, pH_24h_: pH at 24 h after slaughter, DL: drip loss, TL: thaw loss, CL: cooking loss, SF: shear force, L^*^: luminosity (L^*^ = 0 dark meat, L = 100 white meat), a^*^: meat color, ranging from red to green (high a^*^ = red color and low a^*^ = green color), b^*^: meat color, ranging from yellow to blue (high b^*^ = more yellow color and low b^*^ = bluer color), Chroma: color purity.

^3^(1) control diet containing isolated phytase and xylanase valued at 40 kcal of ME/kg (CD0), (2) CD0 + β-mannanase (0.3 g/kg valued at 30 kcal of ME/kg) (CD70), (3) CD0 + β-mannanase (0.3 g/kg valued at 45 kcal of ME/kg) (CD85), and (4) CD0 + β-mannanase (0.3 g/kg valued at 60 kcal of ME/kg) (CD100).

^4^SEM: pooled standard error of the mean.

## 4. Discussion

In the present study, animals were healthy throughout the experiment. However, pigs fed diets supplemented with β-mannanase supported growth performance due to the combined effect of these enzymes in the hydrolysis of antinutritional factors, and as energy sparing and extra energy supply (2, 4, 7). The energy-saving effect of diets supplemented with β-mannanase is attributed to an unnecessary immune deactivation caused by the β-mannans in plant products (2).

A lower SOD concentration is due to the different enzymatic antioxidant system in response to oxidative stress. When pigs are fed diets with reduced ME, the metabolism is changed to use body reserves such as energy and lipids (21). As a result, the process of nutrient oxidation produces energy for animal metabolism; however, energy production is also a source of free radicals (22). In the present study, the greater SOD activity in pigs was performed to eliminate reactive free radicals, as previously reported (23). However, this improved antioxidant capacity did not favor greater energy and nutrient usage in pigs fed CD70 diet.

A previous study (8) reported a higher glucose concentration in pigs fed diets supplemented with β-mannanase-xylanase. The authors mentioned above explained this result based on successful enzyme hydrolysis of NSP, unlike our study, where no differences among the dietary treatments in the biochemical blood profile were observed. The mechanisms of action of these enzymes are supported by the greater usage of hydrolyzed nutrients that favors absorption by the enterocytes in the small intestine (1). However, in the present study, a reduction of 100 kcal of ME/kg diet did not promote greater ATTD in pigs, even with β-mannanase supplementation. This result did not impair the nutrient ATTD coefficients and did not affect the occurrence of intestinal disorders such as diarrhea or increasing digesta viscosity.

On other hand, the results suggested that feeding the CD85 diet to finisher pigs promoted greater DP and DE compared to other dietary treatments, explained by a successful degradation of NSP (8) that improves nutrient usage and energy efficiency due to the increased effectiveness of host enzymes. This mechanism is performed by β-mannanase-xylanase enzymes via breaking down cell walls containing NSP (24) and reducing digesta viscosity (6). However, we did not observe changes on fecal consistency score or the total digesta passage rate in pigs.

Furthermore, β-mannanase supplementation in diets has been previously reported to stimulate the activity of endogenous enzymes (5) and hence favor a greater ATTD in pigs. The effects of phytase on ATTD of nutrients other than calcium and phosphorus have not been well established yet (6). However, the association of dietary xylanase-phytase has been reported to break down cell walls and release more phytic acid to be broken down by phytase (25).

Usually, the consumption of diets with greater energy content promotes increased backfat thickness, as well as the additional energy effect that can be provided by phytase in pig diets (7), which agrees with the results we observed. Pigs fed CD85 diet showed lower backfat thickness measured *in vivo* with ultrasound due to greater energy digestibility even with reduced ME dietary content (8). Although the animals fed CD70 diet had lower DE, the backfat thickness was positively influenced in the animals of this dietary treatment.

Contrary to our observation, enzyme supplementation increased energy digestibility and no effect on backfat thickness in pigs due to dietary energy content was observed in a previous study (24). However, our finding was similar to the one reported by (8), who also observed lower backfat thickness in pigs fed diets supplemented with β-mannanase-xylanase.

Phosphorus and phytate grouped with arabinoxylans have been previously reported (24) to increase redness and reduce water retention in the meat of pigs when exposed to xylanase-phytase action. A similar effect was not observed in the present study; however, we observed dietary treatment effects on pH_24_, which is related to meat quality regarding water retention capacity, color, softness, juiciness, and flavor. Overall, our results agree with those reported by (26), who summarized the higher quality traits of meat from finisher pigs and estimated values of 5.54 for pH_24h_, L^*^ of 46.6, CL of 25.8%, and chroma of 6.2.

In the present study, differences in pH_24h_ among dietary treatments are attributed to muscle glycogen concentration (although not determined in the present study), which largely depends on the diet provided to animals. The lower pH_24h_ value in the meat of pigs fed CD100 diet is related to a greater rate of lactic acid-producing pyruvate, as evidenced by (27). Based on these pH_24h_ values in meat, pigs fed CD0 and CD100 diets showed meats classified as DFD and PSE, respectively (18).

Bacterial diversity in the gastrointestinal tract was assessed in the present study because this is crucial in modulating intestinal functionality and is essential for metabolism, ATTD, and nutrient usage. In general, the balance of the commensal microbiota plays a role in the health of the host. This role is attributed to the diversity of genera and species that possesses protective function, reduces pathogens, inhabit intestinal surfaces, and produces antimicrobial substances (28). The above-mentioned roles attributed to intestinal microbiota promoted animal health during the experimental period.

No effects of dietary treatments on alpha and beta diversity were observed in finisher pigs. This lack of effect could be attributed to the dynamism of microbial communities and different profiles in the gastrointestinal tract segments. In our study, the most prevalent bacterial phyla in pigs were Firmicutes, Proteobacteria, and Bacteroidota, which agrees with previous studies (29, 30). Firmicutes and Bacteroidota are the phyla of greatest representation and paramount importance for gastrointestinal homeostasis (31). An increased incidence of Firmicutes may also be negatively correlated with the presence of potentiality pathogenic bacteria in the intestine of pigs (32). According to (33), a greater presence of organisms of the phylum Firmicutes may create a hostile intestinal environment for pathogenic bacteria colonization.

In a previous study (34), FBR was reported as widely accepted as an evaluative parameter beneficial for intestinal health; therefore, changes in this proportion can trigger several pathologies (35–37). In a study conducted by (38), a greater FBR in pigs was related to improved energy efficiency and growth performance. In addition, similar results were reported in a study conducted on poultry (39); however, these findings differ from our results.

In the present study, the Ruminococcaceae, Lactobacillaceae, and Lachnospiraceae families showed relative abundance in pigs. These families compose the central microbiota of the distal intestine portion and are found in similar proportions in the colon and feces (40). The CD70 diet has positively modulated the growth of Ruminococcaceae family in finisher pigs. This family produces xylanases, cellulases, α-glucosidases, α and β-galactosidases providing greater energy usage (41). In addition, bacteria belonging to the Ruminococcaceae family degrade complex carbohydrates. A reduction in this family has been associated with the use of calorie-rich diets and/or enhanced with carbohydrates (42). This effect could support the reduced Ruminococcaceae occurrence in pigs fed CD0 diet.

Furthermore, no treatment effect on the abundance of Prevotellaceae and Rikenellaceae families was observed in pigs. This finding is associated, in several studies, with a low G:F. Similarly, the Christensenellaceae family was not affected by treatments, which was related to improvement G:F in pigs (43), although a lower G:F was observed in finisher I pigs fed CD0 diet.

However, the Lachnospiraceae family was abundant in the pig gastrointestinal microbiota in both dietary treatments in the present study. This family is known to produce butyric acid (29), which plays a role in maintaining intestinal epithelium structure (41). In addition, the fecal microbiome in finisher pigs showed a relative abundance of families associated with short-chain fatty acids synthesis as final products of sugar fermentation, for example, the Oscillospiraceae (44) and Christensenellaceae families (45).

*Prevotella* is part of the phylum Bacteroidetes, which participates in immune system modulation, metabolic syndromes, and brain-intestine axis regulation (46). This genus was more abundant in pigs fed CD85 diet than in those fed CD100 diet. This result suggests that these changes are related to the different energy content among diets. In fact, *Prevotella* has been reported to play a role in carbohydrate metabolism, such as the degradation of polysaccharides and oligosaccharides usage (47). When analyzed together, part of the modulations observed in pigs occurred in families and genera that play a crucial role in gastrointestinal tract homeostasis.

## 5. Conclusion

Based on the assessing criteria in this study, β-mannanase supplementation in diets containing xylanase-phytase allows reducing 85 kcal of ME/kg because it improves the gain to feed ratio, energy and protein usage, and backfat thickness without metabolic and intestinal ecosystem disorders in finisher pigs. Furthermore, reducing dietary ME alters the fecal microbiome in finisher pigs regardless of the combined enzymes.

## Data availability statement

The original contributions presented in the study are included in the article/supplementary material, further inquiries can be directed to the corresponding author.

## Ethics statement

All experimental procedures performed were approved by the Ethics Committee on the use of production animals at the Universidade Estadual do Oeste do Paraná (Authorization number 17/2022).

## Author contributions

PC, MK, and TP: conceptualization, data curation, and project management. JG, PR, LA, DH, and SC: methodology. JG and NO: software. JG, PR, and NO: statistical analysis, formal analysis, and writing—original draft preparation. PC, SC, and MK: validation. PR, PC, LA, DH, and SC: investigation. JG, PR, MK, GG, HV, and TP: writing—review and editing. PC, SC, AD, and MK: supervision. All authors contributed to the article and approved the submitted version.

## References

[1] Liu S Ma C Liu L Ning D Liu Y Dong B. β-Xylosidase and β-mannosidase in combination improved growth performance and altered microbial profiles in weanling pigs fed a corn-soybean meal-based diet. Asian-Australas J Anim Sci. (2019) 32:1734. 10.5713/ajas.18.0873 31010999 PMC6817776

[2] Vangroenweghe F Poulsen K Thas O. Supplementation of a β-mannanase enzyme reduces post-weaning diarrhea and antibiotic use in piglets on an alternative diet with additional soybean meal. Porc Health Manag. (2021) 7:1–12. 10.1186/s40813-021-00191-5 33431048 PMC7798280

[3] Mok CH Lee JH Kim BG. Effects of exogenous phytase and β-mannanase on ileal and total tract digestibility of energy and nutrient in palm kernel expeller-containing diets fed to growing pigs. Anim Feed Sci Technol. (2013) 186:209–13. 10.1016/j.anifeedsci.2013.10.008

[4] Tiwari UP Chen H Kim SW Jha R. Supplemental effect of xylanase and mannanase on nutrient digestibility and gut health of nursery pigs studied using both *in vivo* and *in vitro* models. Anim Feed Sci Technol. (2018) 245:77–90. 10.1016/j.anifeedsci.2018.07.002

[5] Kipper M Andretta I Quadros VRD Schroeder B Pires PGDS Franceschina CS França I. Performance responses of broilers and pigs fed diets with β-mannanase. Rev Bras Zootec. (2020) 49:1–11. 10.37496/rbz492018017734888489

[6] Yang YY Fan YF Cao YH Guo PP Dong B Ma YX. Effects of exogenous phytase and xylanase, individually or in combination, and pelleting on nutrient digestibility, available energy content of wheat and performance of growing pigs fed wheat-based diets. Asian-Australas J Anim Sci. (2017) 30:57–63. 10.5713/ajas.15.0876 27004820 PMC5205592

[7] Silva CA Callegari MA Dias CP Bridi AM Pierozan CR Foppa L. Increasing doses of phytase from Citrobacter braakii in diets with reduced inorganic phosphorus and calcium improve growth performance and lean meat of growing and finishing pigs. PLoS ONE. (2019) 14:e0217490. 10.1371/journal.pone.0217490 31125379 PMC6534334

[8] Cho JH Kim IH. Effects of beta mannanase and xylanase supplementation in low energy density diets on performances, nutrient digestibility, blood profiles and meat quality in finishing pigs. Asian J Anim Vet Adv. (2013) 8:622–30. 10.3923/ajava.2013.622.630

[9] Rostagno HS Albino LFT Hannas MI Donzele JL Sakomura NK Perazzo FG. Tabelas Brasileiras para aves e suí*nos: composição de alimentos e exigências nutricionais*. Viçosa, MG: UFV (2017).

[10] Silva DJ Queiroz AC. Análises de alimentos (métodos quí*micos e biológicos)*. Viçosa, MG: UFV (2002).

[11] Hart GK Dobb GJ. Effect of a fecal bulking agent on diarrhea during enteral feeding in the critically ill. JPEN J Parenter Enteral Nutr. (1988) 12:465–8. 10.1177/0148607188012005465 3141642

[12] Sakomura NK Rostagno HS. Métodos de pesquisa em nutrição de monogástricos. Jaboticabal, SP: FUNEP (2016).

[13] Kavanagh S Lynch PB O'Mara F Caffrey PJ A. comparison of total collection and marker technique for the measurement of apparent digestibility of diets for growing pigs. Anim Feed Sci Technol. (2001) 89:49–58. 10.1016/S0377-8401(00)00237-6

[14] Owusu-Asiedu AJFJ Patience JF Laarveld B Van Kessel AG Simmins PH Zijlstra RT. Effects of guar gum and cellulose on digesta passage rate, ileal microbial populations, energy and protein digestibility, and performance of grower pigs. J Anim Sci. (2006) 84:843–52. 10.2527/2006.844843x 16543561

[15] Degnan PH Ochman H. Illumina-based analysis of microbial community diversity. ISME J. (2012) 6:183–94. 10.1038/ismej.2011.74 21677692 PMC3246231

[16] Caporaso JG Lauber CL Walters WA Berg-Lyons D Lozupone CA Turnbaugh PJ. Global patterns of 16S rRNA diversity at a depth of millions of sequences per sample. Proc Natl Acad Sci. (2011) 108:4516–22. 10.1073/pnas.1000080107 20534432 PMC3063599

[17] Yilmaz P Parfrey LW Yarza P Gerken J Pruesse E Quast C. The SILVA and “all-species living tree project (LTP)” taxonomic frameworks. Nucleic Acids Res. (2013) 42:D643–48. 10.1093/nar/gkt1209 24293649 PMC3965112

[18] Bridi AM Silva CA. Métodos de avaliação da carcaça e da carne suína. Londrina, PR: Midigraft (2009).

[19] McMurdie PJ Holmes S. phyloseq: an R package for reproducible interactive analysis and graphics of microbiome census data. PLoS One. (2013) 8:e61217. 10.1371/journal.pone.0061217 23630581 PMC3632530

[20] Lahti L, Shetty, S,. Introduction to the Microbiome R Package. (2018). Available online at: https://microbiome.github.io/tutorials/ (Accessed March 15, 2022).

[21] Belhadj Slimen I Najar T Ghram A Abdrrabba M. Heat stress effects on livestock: molecular, cellular and metabolic aspects, a review. J Anim Physiol Anim Nutr. (2016) 100:401–12. 10.1111/jpn.12379 26250521

[22] Celi P Gabai G. Oxidant/antioxidant balance in animal nutrition and health: the role of protein oxidation. Front Vet Sci. (2015) 2:48. 10.3389/fvets.2015.00048 26664975 PMC4672199

[23] Li Z Tang L Liu N Zhang F Liu X Jiang Q. Comparative effects of compound enzyme and antibiotics on growth performance, nutrient digestibility, blood biochemical index, and intestinal health in weaned pigs. Front Microbiol. (2021) 12:768767. 10.3389/fmicb.2021.768767 34777322 PMC8586506

[24] Cho JH Park JH Lee DH Lee JM Song TH Kim IH. Effects of xylanase supplementation on growth performance, digestibility, fecal gas emission, and meat quality in growing–finishing pigs. Can J Anim Sci. (2016) 97:95–100. 10.1139/CJAS-2015-0198

[25] Kim JC Sands JS Mullan BP Pluske JR. Performance and total-tract digestibility responses to exogenous xylanase and phytase in diets for growing pigs. Anim Feed Sci Technol. (2008) 142:163–72. 10.1016/j.anifeedsci.2007.07.004

[26] Sardi L Gastaldo A Borciani M Bertolini A Musi V Garavaldi A. Pre-slaughter sources of fresh meat quality variation: The case of heavy pigs intended for protected designation of origin products. Animals. (2020) 10:2386. 10.3390/ani10122386 33327382 PMC7764830

[27] Yin Y Liu Y Duan G Han M Gong S Yang Z. The effect of dietary leucine supplementation on antioxidant capacity and meat quality of finishing pigs under heat stress. Antioxidants. (2022) 11:1373. 10.3390/antiox11071373 35883864 PMC9312205

[28] Duda-Chodak A Tarko T Satora P Sroka P. Interaction of dietary compounds, especially polyphenols, with the intestinal microbiota: a review. Eur J Nutr. (2015) 54:325–41. 10.1007/s00394-015-0852-y 25672526 PMC4365176

[29] Gresse R Durand FC Dunière L Blanquet-Diot S Forano E. Microbiota composition and functional profiling throughout the gastrointestinal tract of commercial weaning piglets. Microorganisms. (2019) 7:343. 10.3390/microorganisms7090343 31547478 PMC6780805

[30] Kim BR Shin J Guevarra RB Lee JH Kim DW Seol KH. Deciphering diversity indices for a better understanding of microbial communities. J Microbiol Biotechnol. (2017) 27:2089–93. 10.4014/jmb.1709.09027 29032640

[31] Rychlik I. Composition and function of chicken gut microbiota. Animals. (2020) 10:103. 10.3390/ani10010103 31936291 PMC7022619

[32] Mulder IE Schmidt B Stokes CR Lewis M Bailey M Aminov RI. Environmentally-acquired bacteria influence microbial diversity and natural innate immune responses at gut surfaces. BMC Biol. (2009) 7:1–20. 10.1186/1741-7007-7-79 19930542 PMC2785767

[33] Molist F Manzanilla EG Pérez JF Nyachoti CM. Coarse, but not finelyground, dietary fibre increases intestinal firmicutes:bacteroidetes ratio and reduces diarrhoea induced by experimental infection in piglets. Br J Nutr. (2012) 108:9–15. 10.1017/S0007114511005216 22018207

[34] Wang Z Tang Y Long L Zhang H. Effects of dietary L-theanine on growth performance, antioxidation, meat quality and intestinal microflora in white feather broilers with acute oxidative stress. Front Vet Sci. (2022) 696: 889485. 10.3389/fvets.2022.889485 35812843 PMC9267357

[35] Magne F Gotteland M Gauthier L Zazueta A Pesoa S Navarrete P Balamurugan R. The firmicutes/bacteroidetes ratio: a relevant marker of gut dysbiosis in obese patients? Nutrients. (2020) 12:1474. 10.3390/nu12051474 32438689 PMC7285218

[36] Stojanov S Berlec A Štrukelj B. The influence of probiotics on the firmicutes/bacteroidetes ratio in the treatment of obesity and inflammatory bowel disease. Microorganisms. (2020) 8:1–16. 10.3390/microorganisms8111715 33139627 PMC7692443

[37] Turnbaugh PJ Ley RE Mahowald MA Magrini V Mardis ER Gordon JI. An obesity-associated gut microbiome with increased capacity for energy harvest. Nature. (2006) 444:1027–31. 10.1038/nature05414 17183312

[38] Zhao W Wang Y Liu S Huang J Zhai Z He C. The dynamic distribution of porcine microbiota across different ages and gastrointestinal tract segments. PLoS ONE. (2015) 10:1–13. 10.1371/journal.pone.0117441 25688558 PMC4331431

[39] Xu Y Yang H Zhang L Su Y Shi D Xiao H. High-through put sequencing technology to reveal the composition and function of cecal microbiota in Dagu chicken. BMC Microbiol. (2016) 16:1–9. 10.1186/s12866-016-0877-2 27814685 PMC5097418

[40] Gierse LC Meene A Schultz D Schwaiger T Karte C Schröder C. A multi-omics protocol for swine feces to elucidate longitudinal dynamics in microbiome structure and function. Microorganisms. (2020) 8:1–20. 10.3390/microorganisms8121887 33260576 PMC7760263

[41] Biddle A Stewart L Blanchard J Leschine S. Untangling the genetic basis of fibrolytic specialization by Lachnospiraceae and Ruminococcaceae in diverse gut communities. Diversity. (2013) 5:627–40. 10.3390/d5030627

[42] Lagkouvardos I Lesker TR Hitch TCA Gálvez EJC Smit N Neuhaus K. Sequence and cultivation study of Muribaculaceae reveals novel species, host preference, and functional potential of this yet undescribed family. Microbiome. (2019) 7:28. 10.1186/s40168-019-0637-2 30782206 PMC6381624

[43] Quan J Cai G Ye J Yang M Ding R Wang X. A global comparison of the microbiome compositions of three gut locations in commercial pigs with extreme feed conversion ratios. Sci Rep. (2018) 8:4536. 10.1038/s41598-018-22692-0 29540768 PMC5852056

[44] Beaumont M Cauquil L Bertide A Ahn I Barilly C Gil L. Gut microbiota-derived metabolite signature in suckling and weaned piglets. J Proteome Res. (2021) 20:982–94. 10.1021/acs.jproteome.0c00745 33289566

[45] Morotomi M Nagai F Watanabe Y. Description of Christensenella minutagen. Nov., sp nov, isolated from human faeces, which forms a distinct branch in the order Clostridiales, and proposal of Christensenellaceae fam nov. Int J Syst Evol Microbiol. (2012) 62:144–9. 10.1099/ijs.0.026989-0 21357455

[46] Gibiino G Lopetuso LR Scaldaferri F Rizzatti G Binda C Gasbarrini A. Exploring bacteroidetes: metabolic key points and immunological tricks of our gut commensals. Dig Liver Dis. (2018) 50:635–9. 10.1016/j.dld.2018.03.016 29650468

[47] Zhang D Liu H Wang S Zhang W Wang J Tian H. Fecal microbiota and its correlation with fatty acids and free amino acids metabolism in piglets after a Lactobacillus strain oral administration. Front Microbiol. (2019) 10:785. 10.3389/fmicb.2019.00785 31040835 PMC6476935

